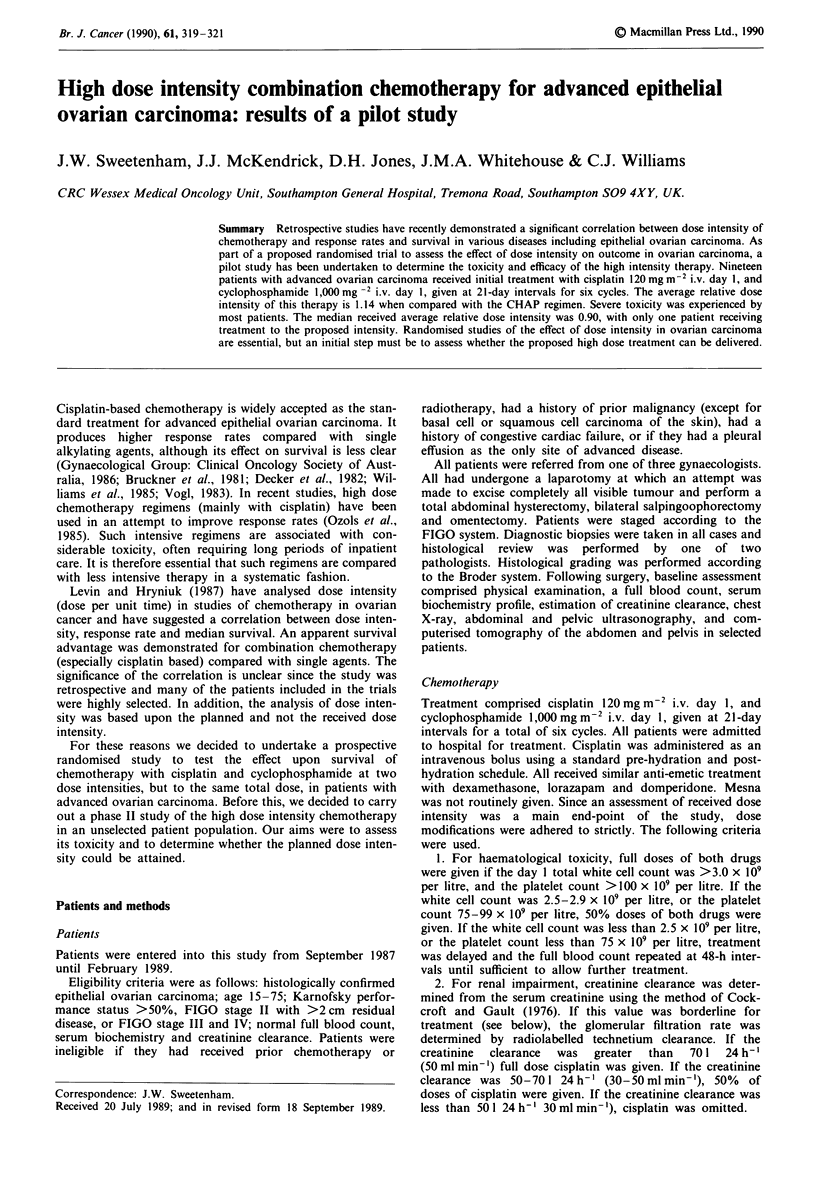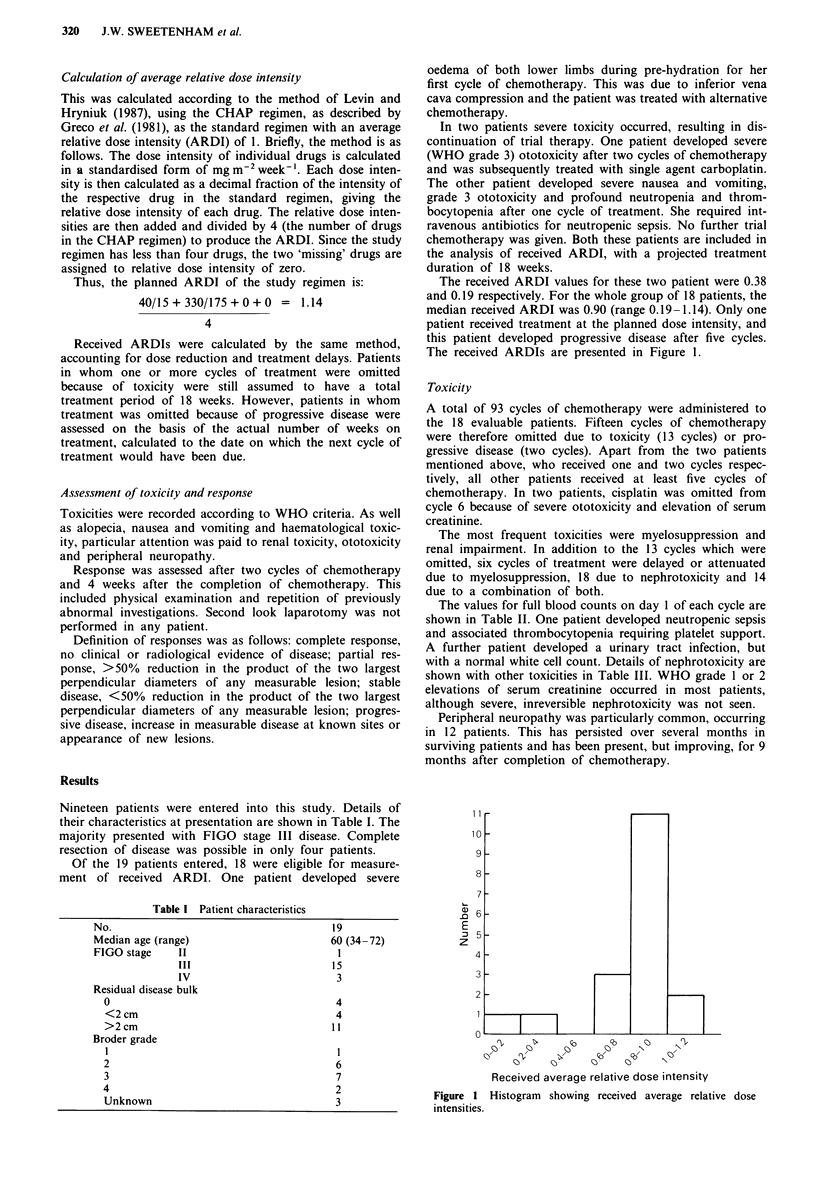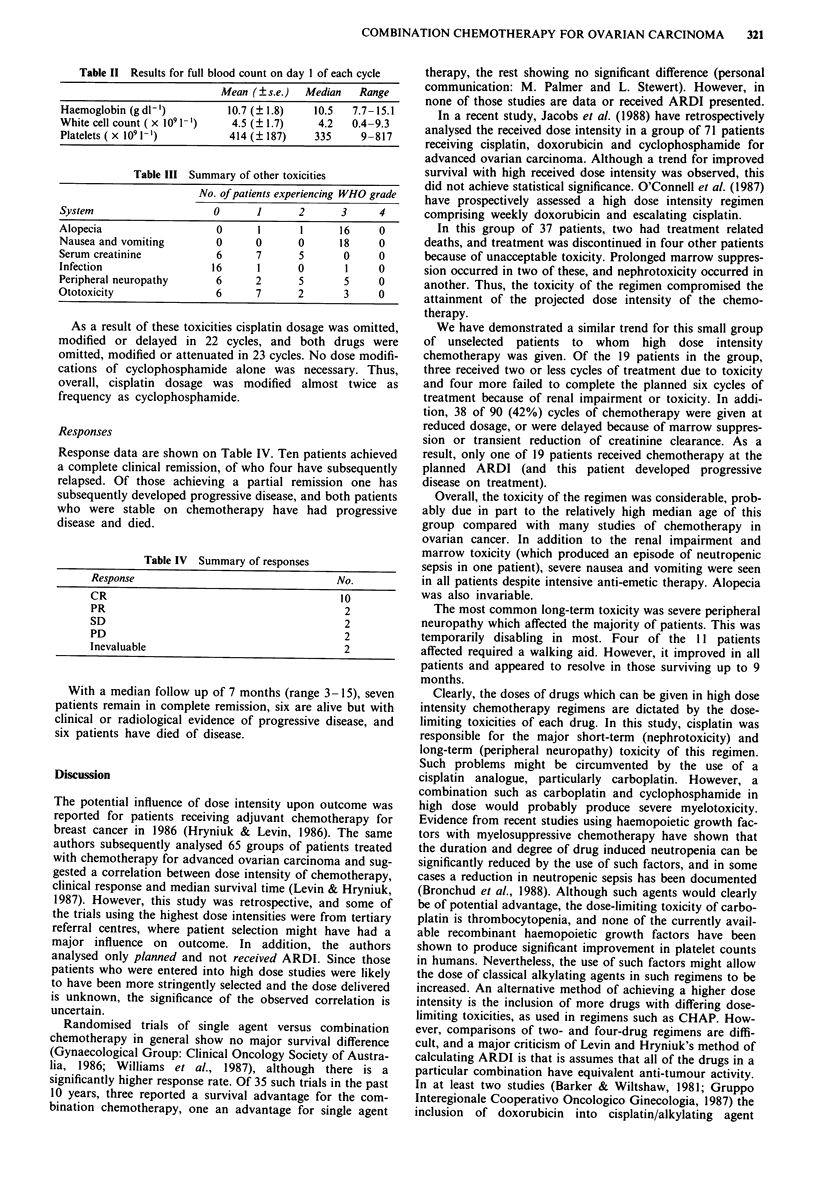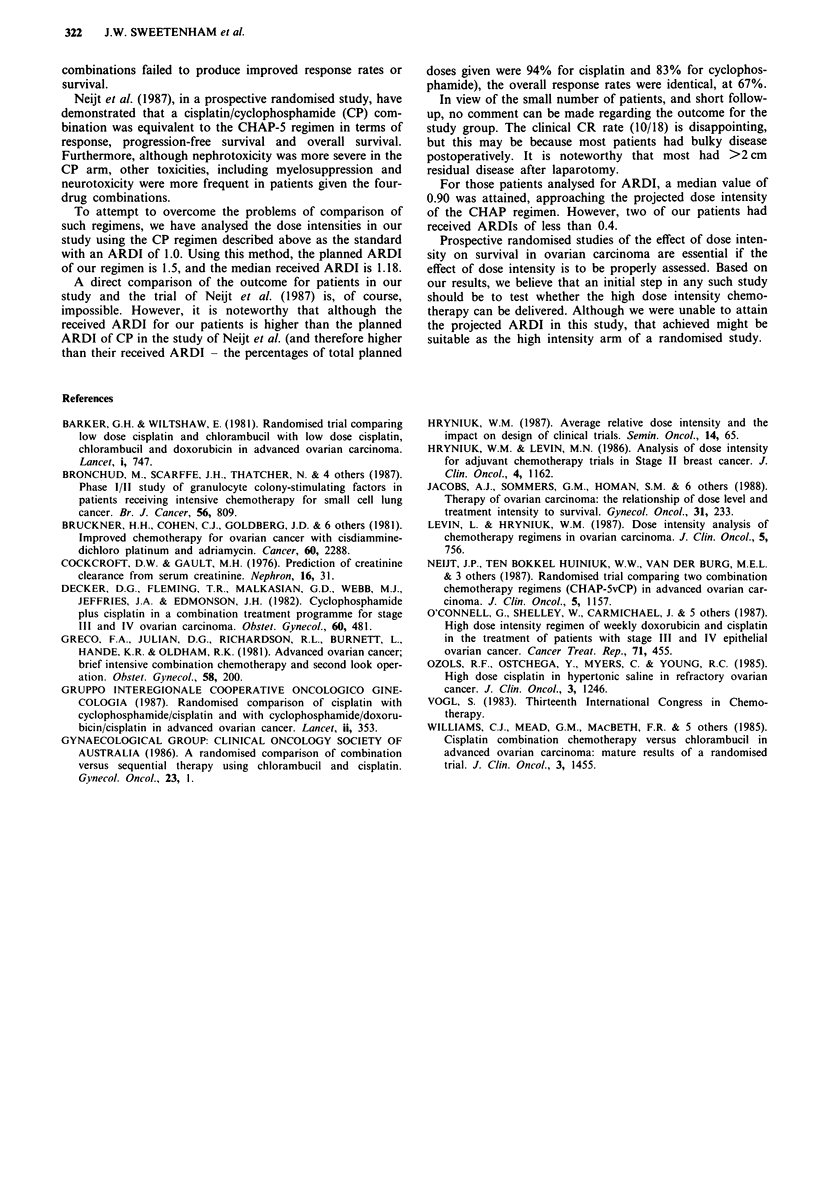# High dose intensity combination chemotherapy for advanced epithelial ovarian carcinoma: results of a pilot study.

**DOI:** 10.1038/bjc.1990.61

**Published:** 1990-02

**Authors:** J. W. Sweetenham, J. J. McKendrick, D. H. Jones, J. M. Whitehouse, C. J. Williams

**Affiliations:** CRC Wessex Medical Oncology Unit, Southampton General Hospital, UK.

## Abstract

Retrospective studies have recently demonstrated a significant correlation between dose intensity of chemotherapy and response rates and survival in various diseases including epithelial ovarian carcinoma. As part of a proposed randomised trial to assess the effect of dose intensity on outcome in ovarian carcinoma, a pilot study has been undertaken to determine the toxicity and efficacy of the high intensity therapy. Nineteen patients with advanced ovarian carcinoma received initial treatment with cisplatin 120 mg m-2 i.v. day 1, and cyclophosphamide 1,000 mg-2 i.v. day 1, given at 21-day intervals for six cycles. The average relative dose intensity of this therapy is 1.14 when compared with the CHAP regimen. Severe toxicity was experienced by most patients. The median received average relative dose intensity was 0.90, with only one patient receiving treatment to the proposed intensity. Randomised studies of the effect of dose intensity in ovarian carcinoma are essential, but an initial step must be to assess whether the proposed high dose treatment can be delivered.


Br. J. Cancer (1990), 61, 319-321

© Macmillan Press Ltd., 1990

### High dose intensity combination chemotherapy for advanced epithelial ovarian carcinoma: results of a pilot study

J.W. Sweetenham, J.J. McKendrick, D.H. Jones, J.M.A. Whitehouse & C.J. Williams

*CRC Wessex Medical Oncology Unit, Southampton General Hospital, Tremona Road, Southampton SO9 4XY, UK.*

**Summary** Retrospective studies have recently demonstrated a significant correlation between dose intensity of chemotherapy and response rates and survival in various diseases including epithelial ovarian carcinoma. As part of a proposed randomised trial to assess the effect of dose intensity on outcome in ovarian carcinoma, a pilot study has been undertaken to determine the toxicity and efficacy of the high intensity therapy. Nineteen patients with advanced ovarian carcinoma received initial treatment with cisplatin 120 mg $m^{-2}$ i.v. day 1, and cyclophosphamide 1,000 mg $^{-2}$ i.v. day 1, given at 21-day intervals for six cycles. The average relative dose intensity of this therapy is 1.14 when compared with the CHAP regimen. Severe toxicity was experienced by most patients. The median received average relative dose intensity was 0.90, with only one patient receiving treatment to the proposed intensity. Randomised studies of the effect of dose intensity in ovarian carcinoma are essential, but an initial step must be to assess whether the proposed high dose treatment can be delivered.

Cisplatin-based chemotherapy is widely accepted as the standard treatment for advanced epithelial ovarian carcinoma. It produces higher response rates compared with single alkylating agents, although its effect on survival is less clear (Gynaecological Group: Clinical Oncology Society of Australia, 1986; Bruckner *et al.*, 1981; Decker *et al.*, 1982; Williams *et al.*, 1985; Vogl, 1983). In recent studies, high dose chemotherapy regimens (mainly with cisplatin) have been used in an attempt to improve response rates (Ozols *et al.*, 1985). Such intensive regimens are associated with considerable toxicity, often requiring long periods of inpatient care. It is therefore essential that such regimens are compared with less intensive therapy in a systematic fashion.

Levin and Hryniuk (1987) have analysed dose intensity (dose per unit time) in studies of chemotherapy in ovarian cancer and have suggested a correlation between dose intensity, response rate and median survival. An apparent survival advantage was demonstrated for combination chemotherapy (especially cisplatin based) compared with single agents. The significance of the correlation is unclear since the study was retrospective and many of the patients included in the trials were highly selected. In addition, the analysis of dose intensity was based upon the planned and not the received dose intensity.

For these reasons we decided to undertake a prospective randomised study to test the effect upon survival of chemotherapy with cisplatin and cyclophosphamide at two dose intensities, but to the same total dose, in patients with advanced ovarian carcinoma. Before this, we decided to carry out a phase II study of the high dose intensity chemotherapy in an unselected patient population. Our aims were to assess its toxicity and to determine whether the planned dose intensity could be attained.

### Patients and methods

### *Patients*

Patients were entered into this study from September 1987 until February 1989.

Eligibility criteria were as follows: histologically confirmed epithelial ovarian carcinoma; age 15-75; Karnofsky performance status >50%, FIGO stage II with >2 cm residual disease, or FIGO stage III and IV; normal full blood count, serum biochemistry and creatinine clearance. Patients were ineligible if they had received prior chemotherapy or radiotherapy, had a history of prior malignancy (except for basal cell or squamous cell carcinoma of the skin), had a history of congestive cardiac failure, or if they had a pleural effusion as the only site of advanced disease.

All patients were referred from one of three gynaecologists. All had undergone a laparotomy at which an attempt was made to excise completely all visible tumour and perform a total abdominal hysterectomy, bilateral salpingoophorectomy and omentectomy. Patients were staged according to the FIGO system. Diagnostic biopsies were taken in all cases and histological review was performed by one of two pathologists. Histological grading was performed according to the Broder system. Following surgery, baseline assessment comprised physical examination, a full blood count, serum biochemistry profile, estimation of creatinine clearance, chest X-ray, abdominal and pelvic ultrasonography, and computerised tomography of the abdomen and pelvis in selected patients.

### *Chemotherapy*

Treatment comprised cisplatin 120 mg $m^{-2}$ i.v. day 1, and cyclophosphamide 1,000 mg $m^{-2}$ i.v. day 1, given at 21-day intervals for a total of six cycles. All patients were admitted to hospital for treatment. Cisplatin was administered as an intravenous bolus using a standard pre-hydration and post-hydration schedule. All received similar anti-emetic treatment with dexamethasone, lorazapam and domperidone. Mesna was not routinely given. Since an assessment of received dose intensity was a main end-point of the study, dose modifications were adhered to strictly. The following criteria were used.

1. For haematological toxicity, full doses of both drugs were given if the day 1 total white cell count was $>3.0 \times 10^9$ per litre, and the platelet count $>100 \times 10^9$ per litre. If the white cell count was $2.5-2.9 \times 10^9$ per litre, or the platelet count $75-99 \times 10^9$ per litre, 50% doses of both drugs were given. If the white cell count was less than $2.5 \times 10^9$ per litre, or the platelet count less than $75 \times 10^9$ per litre, treatment was delayed and the full blood count repeated at 48-h intervals until sufficient to allow further treatment.

2. For renal impairment, creatinine clearance was determined from the serum creatinine using the method of Cockcroft and Gault (1976). If this value was borderline for treatment (see below), the glomerular filtration rate was determined by radiolabelled technetium clearance. If the creatinine clearance was greater than 70 l 24 $h^{-1}$ (50 ml $min^{-1}$) full dose cisplatin was given. If the creatinine clearance was 50-70 l 24 $h^{-1}$ (30-50 ml $min^{-1}$), 50% of doses of cisplatin were given. If the creatinine clearance was less than 50 l 24 $h^{-1}$ 30 ml $min^{-1}$), cisplatin was omitted.

---

Correspondence: J.W. Sweetenham.
Received 20 July 1989; and in revised form 18 September 1989.

*Calculation of average relative dose intensity*

This was calculated according to the method of Levin and Hryniuk (1987), using the CHAP regimen, as described by Greco *et al.* (1981), as the standard regimen with an average relative dose intensity (ARDI) of 1. Briefly, the method is as follows. The dose intensity of individual drugs is calculated in a standardised form of mg $m^{-2}$ $week^{-1}$. Each dose intensity is then calculated as a decimal fraction of the intensity of the respective drug in the standard regimen, giving the relative dose intensity of each drug. The relative dose intensities are then added and divided by 4 (the number of drugs in the CHAP regimen) to produce the ARDI. Since the study regimen has less than four drugs, the two 'missing' drugs are assigned to relative dose intensity of zero.

Thus, the planned ARDI of the study regimen is:

$$\frac{40/15 + 330/175 + 0 + 0}{4} = 1.14$$

Received ARDIs were calculated by the same method, accounting for dose reduction and treatment delays. Patients in whom one or more cycles of treatment were omitted because of toxicity were still assumed to have a total treatment period of 18 weeks. However, patients in whom treatment was omitted because of progressive disease were assessed on the basis of the actual number of weeks on treatment, calculated to the date on which the next cycle of treatment would have been due.

*Assessment of toxicity and response*

Toxicities were recorded according to WHO criteria. As well as alopecia, nausea and vomiting and haematological toxicity, particular attention was paid to renal toxicity, ototoxicity and peripheral neuropathy.

Response was assessed after two cycles of chemotherapy and 4 weeks after the completion of chemotherapy. This included physical examination and repetition of previously abnormal investigations. Second look laparotomy was not performed in any patient.

Definition of responses was as follows: complete response, no clinical or radiological evidence of disease; partial response, >50% reduction in the product of the two largest perpendicular diameters of any measurable lesion; stable disease, <50% reduction in the product of the two largest perpendicular diameters of any measurable lesion; progressive disease, increase in measurable disease at known sites or appearance of new lesions.

### Results

Nineteen patients were entered into this study. Details of their characteristics at presentation are shown in Table I. The majority presented with FIGO stage III disease. Complete resection of disease was possible in only four patients.

Of the 19 patients entered, 18 were eligible for measurement of received ARDI. One patient developed severe oedema of both lower limbs during pre-hydration for her first cycle of chemotherapy. This was due to inferior vena cava compression and the patient was treated with alternative chemotherapy.

**Table I** Patient characteristics

| | | |
|---|---|---|
| No. | | 19 |
| Median age (range) | | 60 (34–72) |
| FIGO stage | II | 1 |
| | III | 15 |
| | IV | 3 |
| Residual disease bulk | | |
| 0 | | 4 |
| <2 cm | | 4 |
| >2 cm | | 11 |
| Broder grade | | |
| 1 | | 1 |
| 2 | | 6 |
| 3 | | 7 |
| 4 | | 2 |
| Unknown | | 3 |

In two patients severe toxicity occurred, resulting in discontinuation of trial therapy. One patient developed severe (WHO grade 3) ototoxicity after two cycles of chemotherapy and was subsequently treated with single agent carboplatin. The other patient developed severe nausea and vomiting, grade 3 ototoxicity and profound neutropenia and thrombocytopenia after one cycle of treatment. She required intravenous antibiotics for neutropenic sepsis. No further trial chemotherapy was given. Both these patients are included in the analysis of received ARDI, with a projected treatment duration of 18 weeks.

The received ARDI values for these two patient were 0.38 and 0.19 respectively. For the whole group of 18 patients, the median received ARDI was 0.90 (range 0.19–1.14). Only one patient received treatment at the planned dose intensity, and this patient developed progressive disease after five cycles. The received ARDIs are presented in Figure 1.

*Toxicity*

A total of 93 cycles of chemotherapy were administered to the 18 evaluable patients. Fifteen cycles of chemotherapy were therefore omitted due to toxicity (13 cycles) or progressive disease (two cycles). Apart from the two patients mentioned above, who received one and two cycles respectively, all other patients received at least five cycles of chemotherapy. In two patients, cisplatin was omitted from cycle 6 because of severe ototoxicity and elevation of serum creatinine.

The most frequent toxicities were myelosuppression and renal impairment. In addition to the 13 cycles which were omitted, six cycles of treatment were delayed or attenuated due to myelosuppression, 18 due to nephrotoxicity and 14 due to a combination of both.

The values for full blood counts on day 1 of each cycle are shown in Table II. One patient developed neutropenic sepsis and associated thrombocytopenia requiring platelet support. A further patient developed a urinary tract infection, but with a normal white cell count. Details of nephrotoxicity are shown with other toxicities in Table III. WHO grade 1 or 2 elevations of serum creatinine occurred in most patients, although severe, inreversible nephrotoxicity was not seen.

Peripheral neuropathy was particularly common, occurring in 12 patients. This has persisted over several months in surviving patients and has been present, but improving, for 9 months after completion of chemotherapy.

**Figure 1** Histogram showing received average relative dose intensities.

**Table II** Results for full blood count on day 1 of each cycle

| | *Mean (±s.e.)* | *Median* | *Range* |
|---|---|---|---|
| Haemoglobin (g $dl^{-1}$) | 10.7 (±1.8) | 10.5 | 7.7–15.1 |
| White cell count ( × $10^9 l^{-1}$) | 4.5 (±1.7) | 4.2 | 0.4–9.3 |
| Platelets ( × $10^9 l^{-1}$) | 414 (±187) | 335 | 9–817 |

**Table III** Summary of other toxicities

| | *No. of patients experiencing WHO grade* | | | | |
|---|---|---|---|---|---|
| *System* | *0* | *1* | *2* | *3* | *4* |
| Alopecia | 0 | 1 | 1 | 16 | 0 |
| Nausea and vomiting | 0 | 0 | 0 | 18 | 0 |
| Serum creatinine | 6 | 7 | 5 | 0 | 0 |
| Infection | 16 | 1 | 0 | 1 | 0 |
| Peripheral neuropathy | 6 | 2 | 5 | 5 | 0 |
| Ototoxicity | 6 | 7 | 2 | 3 | 0 |

As a result of these toxicities cisplatin dosage was omitted, modified or delayed in 22 cycles, and both drugs were omitted, modified or attenuated in 23 cycles. No dose modifications of cyclophosphamide alone was necessary. Thus, overall, cisplatin dosage was modified almost twice as frequency as cyclophosphamide.

*Responses*

Response data are shown on Table IV. Ten patients achieved a complete clinical remission, of who four have subsequently relapsed. Of those achieving a partial remission one has subsequently developed progressive disease, and both patients who were stable on chemotherapy have had progressive disease and died.

**Table IV** Summary of responses

| *Response* | *No.* |
|---|---|
| CR | 10 |
| PR | 2 |
| SD | 2 |
| PD | 2 |
| Inevaluable | 2 |

With a median follow up of 7 months (range 3–15), seven patients remain in complete remission, six are alive but with clinical or radiological evidence of progressive disease, and six patients have died of disease.

### Discussion

The potential influence of dose intensity upon outcome was reported for patients receiving adjuvant chemotherapy for breast cancer in 1986 (Hryniuk & Levin, 1986). The same authors subsequently analysed 65 groups of patients treated with chemotherapy for advanced ovarian carcinoma and suggested a correlation between dose intensity of chemotherapy, clinical response and median survival time (Levin & Hryniuk, 1987). However, this study was retrospective, and some of the trials using the highest dose intensities were from tertiary referral centres, where patient selection might have had a major influence on outcome. In addition, the authors analysed only *planned* and not *received* ARDI. Since those patients who were entered into high dose studies were likely to have been more stringently selected and the dose delivered is unknown, the significance of the observed correlation is uncertain.

Randomised trials of single agent versus combination chemotherapy in general show no major survival difference (Gynaecological Group: Clinical Oncology Society of Australia, 1986; Williams *et al.*, 1987), although there is a significantly higher response rate. Of 35 such trials in the past 10 years, three reported a survival advantage for the combination chemotherapy, one an advantage for single agent therapy, the rest showing no significant difference (personal communication: M. Palmer and L. Stewert). However, in none of those studies are data or received ARDI presented.

In a recent study, Jacobs *et al.* (1988) have retrospectively analysed the received dose intensity in a group of 71 patients receiving cisplatin, doxorubicin and cyclophosphamide for advanced ovarian carcinoma. Although a trend for improved survival with high received dose intensity was observed, this did not achieve statistical significance. O'Connell *et al.* (1987) have prospectively assessed a high dose intensity regimen comprising weekly doxorubicin and escalating cisplatin.

In this group of 37 patients, two had treatment related deaths, and treatment was discontinued in four other patients because of unacceptable toxicity. Prolonged marrow suppression occurred in two of these, and nephrotoxicity occurred in another. Thus, the toxicity of the regimen compromised the attainment of the projected dose intensity of the chemotherapy.

We have demonstrated a similar trend for this small group of unselected patients to whom high dose intensity chemotherapy was given. Of the 19 patients in the group, three received two or less cycles of treatment due to toxicity and four more failed to complete the planned six cycles of treatment because of renal impairment or toxicity. In addition, 38 of 90 (42%) cycles of chemotherapy were given at reduced dosage, or were delayed because of marrow suppression or transient reduction of creatinine clearance. As a result, only one of 19 patients received chemotherapy at the planned ARDI (and this patient developed progressive disease on treatment).

Overall, the toxicity of the regimen was considerable, probably due in part to the relatively high median age of this group compared with many studies of chemotherapy in ovarian cancer. In addition to the renal impairment and marrow toxicity (which produced an episode of neutropenic sepsis in one patient), severe nausea and vomiting were seen in all patients despite intensive anti-emetic therapy. Alopecia was also invariable.

The most common long-term toxicity was severe peripheral neuropathy which affected the majority of patients. This was temporarily disabling in most. Four of the 11 patients affected required a walking aid. However, it improved in all patients and appeared to resolve in those surviving up to 9 months.

Clearly, the doses of drugs which can be given in high dose intensity chemotherapy regimens are dictated by the dose-limiting toxicities of each drug. In this study, cisplatin was responsible for the major short-term (nephrotoxicity) and long-term (peripheral neuropathy) toxicity of this regimen. Such problems might be circumvented by the use of a cisplatin analogue, particularly carboplatin. However, a combination such as carboplatin and cyclophosphamide in high dose would probably produce severe myelotoxicity. Evidence from recent studies using haemopoietic growth factors with myelosuppressive chemotherapy have shown that the duration and degree of drug induced neutropenia can be significantly reduced by the use of such factors, and in some cases a reduction in neutropenic sepsis has been documented (Bronchud *et al.*, 1988). Although such agents would clearly be of potential advantage, the dose-limiting toxicity of carboplatin is thrombocytopenia, and none of the currently available recombinant haemopoietic growth factors have been shown to produce significant improvement in platelet counts in humans. Nevertheless, the use of such factors might allow the dose of classical alkylating agents in such regimens to be increased. An alternative method of achieving a higher dose intensity is the inclusion of more drugs with differing dose-limiting toxicities, as used in regimens such as CHAP. However, comparisons of two- and four-drug regimens are difficult, and a major criticism of Levin and Hryniuk's method of calculating ARDI is that is assumes that all of the drugs in a particular combination have equivalent anti-tumour activity. In at least two studies (Barker & Wiltshaw, 1981; Gruppo Interegionale Cooperativo Oncologico Ginecologia, 1987) the inclusion of doxorubicin into cisplatin/alkylating agent combinations failed to produce improved response rates or survival.

Neijt *et al.* (1987), in a prospective randomised study, have demonstrated that a cisplatin/cyclophosphamide (CP) combination was equivalent to the CHAP-5 regimen in terms of response, progression-free survival and overall survival. Furthermore, although nephrotoxicity was more severe in the CP arm, other toxicities, including myelosuppression and neurotoxicity were more frequent in patients given the four-drug combinations.

To attempt to overcome the problems of comparison of such regimens, we have analysed the dose intensities in our study using the CP regimen described above as the standard with an ARDI of 1.0. Using this method, the planned ARDI of our regimen is 1.5, and the median received ARDI is 1.18.

A direct comparison of the outcome for patients in our study and the trial of Neijt *et al.* (1987) is, of course, impossible. However, it is noteworthy that although the received ARDI for our patients is higher than the planned ARDI of CP in the study of Neijt *et al.* (and therefore higher than their received ARDI – the percentages of total planned doses given were 94% for cisplatin and 83% for cyclophosphamide), the overall response rates were identical, at 67%.

In view of the small number of patients, and short follow-up, no comment can be made regarding the outcome for the study group. The clinical CR rate (10/18) is disappointing, but this may be because most patients had bulky disease postoperatively. It is noteworthy that most had >2 cm residual disease after laparotomy.

For those patients analysed for ARDI, a median value of 0.90 was attained, approaching the projected dose intensity of the CHAP regimen. However, two of our patients had received ARDIs of less than 0.4.

Prospective randomised studies of the effect of dose intensity on survival in ovarian carcinoma are essential if the effect of dose intensity is to be properly assessed. Based on our results, we believe that an initial step in any such study should be to test whether the high dose intensity chemotherapy can be delivered. Although we were unable to attain the projected ARDI in this study, that achieved might be suitable as the high intensity arm of a randomised study.

### References

BARKER, G.H. & WILTSHAW, E. (1981). Randomised trial comparing low dose cisplatin and chlorambucil with low dose cisplatin, chlorambucil and doxorubicin in advanced ovarian carcinoma. *Lancet*, **i**, 747.

BRONCHUD, M., SCARFFE, J.H., THATCHER, N. & 4 others (1987). Phase I/II study of granulocyte colony-stimulating factors in patients receiving intensive chemotherapy for small cell lung cancer. *Br. J. Cancer*, **56**, 809.

BRUCKNER, H.H., COHEN, C.J., GOLDBERG, J.D. & 6 others (1981). Improved chemotherapy for ovarian cancer with cisdiammine-dichloro platinum and adriamycin. *Cancer*, **60**, 2288.

COCKCROFT, D.W. & GAULT, M.H. (1976). Prediction of creatinine clearance from serum creatinine. *Nephron*, **16**, 31.

DECKER, D.G., FLEMING, T.R., MALKASIAN, G.D., WEBB, M.J., JEFFRIES, J.A. & EDMONSON, J.H. (1982). Cyclophosphamide plus cisplatin in a combination treatment programme for stage III and IV ovarian carcinoma. *Obstet. Gynecol.*, **60**, 481.

GRECO, F.A., JULIAN, D.G., RICHARDSON, R.L., BURNETT, L., HANDE, K.R. & OLDHAM, R.K. (1981). Advanced ovarian cancer; brief intensive combination chemotherapy and second look operation. *Obstet. Gynecol.*, **58**, 200.

GRUPPO INTEREGIONALE COOPERATIVE ONCOLOGICO GINECOLOGIA (1987). Randomised comparison of cisplatin with cyclophosphamide/cisplatin and with cyclophosphamide/doxorubicin/cisplatin in advanced ovarian cancer. *Lancet*, **ii**, 353.

GYNAECOLOGICAL GROUP: CLINICAL ONCOLOGY SOCIETY OF AUSTRALIA (1986). A randomised comparison of combination versus sequential therapy using chlorambucil and cisplatin. *Gynecol. Oncol.*, **23**, 1.

HRYNIUK, W.M. (1987). Average relative dose intensity and the impact on design of clinical trials. *Semin. Oncol.*, **14**, 65.

HRYNIUK, W.M. & LEVIN, M.N. (1986). Analysis of dose intensity for adjuvant chemotherapy trials in Stage II breast cancer. *J. Clin. Oncol.*, **4**, 1162.

JACOBS, A.J., SOMMERS, G.M., HOMAN, S.M. & 6 others (1988). Therapy of ovarian carcinoma: the relationship of dose level and treatment intensity to survival. *Gynecol. Oncol.*, **31**, 233.

LEVIN, L. & HRYNIUK, W.M. (1987). Dose intensity analysis of chemotherapy regimens in ovarian carcinoma. *J. Clin. Oncol.*, **5**, 756.

NEIJT, J.P., TEN BOKKEL HUINIUK, W.W., VAN DER BURG, M.E.L. & 3 others (1987). Randomised trial comparing two combination chemotherapy regimens (CHAP-5vCP) in advanced ovarian carcinoma. *J. Clin. Oncol.*, **5**, 1157.

O'CONNELL, G., SHELLEY, W., CARMICHAEL, J. & 5 others (1987). High dose intensity regimen of weekly doxorubicin and cisplatin in the treatment of patients with stage III and IV epithelial ovarian cancer. *Cancer Treat. Rep.*, **71**, 455.

OZOLS, R.F., OSTCHEGA, Y., MYERS, C. & YOUNG, R.C. (1985). High dose cisplatin in hypertonic saline in refractory ovarian cancer. *J. Clin. Oncol.*, **3**, 1246.

VOGL, S. (1983). Thirteenth International Congress in Chemotherapy.

WILLIAMS, C.J., MEAD, G.M., MACBETH, F.R. & 5 others (1985). Cisplatin combination chemotherapy versus chlorambucil in advanced ovarian carcinoma: mature results of a randomised trial. *J. Clin. Oncol.*, **3**, 1455.